# Moving targets: The challenges of studying infectious diseases among pregnant women in resource limited settings

**DOI:** 10.1016/j.vaccine.2015.08.042

**Published:** 2015-08-25

**Authors:** Titus H. Divala, Randy G. Mungwira, Miriam K. Laufer

**Affiliations:** a Blantyre Malaria Project, University of Malawi College of Medicine, Blantyre, Malawi; b Institute for Global Health, University of Maryland School of Medicine, Baltimore, MD, USA

**Keywords:** Clinical trials, Pregnancy, Infectious diseases, Antenatal care, Generalizability, International health

## Abstract

Conducting clinical trials to prevent and treat infectious diseases in pregnancy is essential to saving maternal and newborn lives, though it is fraught with challenges. We have been conducting research in malaria treatment and prevention in children and pregnant women in Blantyre, Malawi for over a decade. Here, we review some of the unique challenges that we have faced in leading research studies that with rigor and integrity and maintaining the highest ethical standard. We conclude with concrete strategies to overcome some of the apparent obstacles that frequently focus on building trust through bidirectional communication with local health workers and communities. We also highlight the key role of local and international investigators to advocate for the health of the communities in which they work.

## 1. Introduction

Most clinical researchers would have had the experience of a disease sharply decreasing in prevalence, or even virtually disappearing, while the disease is under study. The conditions of a clinical trial often alter the natural history of diseases and the prevalence of adverse outcomes. These complicating factors limit the ability to conduct ethical studies that provide generalizable information, especially in resource-limited settings, where the reality of access to health care and disease prevention is often much less than the stated standard of care [1]. In addition, it contributes to the failure to conduct adequately powered studies to detect differences between treatment and control groups because the overall rate of either the disease or adverse outcome of interest decreases significantly due to the conditions of the clinical trial.

The challenges of conducting clinical studies among pregnant women have been well articulated in previous reviews [2,3]. Our review focuses on unique challenges of clinical trials in resource limited settings using illustrative examples; our research group has encountered conducting studies to evaluate strategies to prevent and treat malaria among pregnant women in Malawi. We highlight common issues that would apply to a wide range of diseases. We end the discussion with several key lessons learned from our experiences and strategies that have overcome some of the challenges that we and others have faced. This discussion is not intended to be exhaustive but rather a framework in which to consider and trouble-shoot the unique obstacles.

## 2. Typical scenario

In clinical trials, to treat or prevent infectious diseases in pregnant women, the study design is typical. Pregnant women at risk of an infectious disease are enrolled at a standardized and often early point in their pregnancy when they are assumed to be uninfected. At baseline, all participants are expected to be at a similar risk of an incident infection over the course of their participation. They are randomized to receive either the intervention or a place boor standard of care. The primary aim is to measure the effect of the intervention on the incidence of an infection during pregnancy or in the infant or on the cure rate. The additional key aim is often to assess the safety of the intervention by measuring its impact on maternal, perinatal and fetal outcomes.

Prospective participants undergo a screening process to ensure that they meet specified eligibility criteria and to exclude women who may be at increased risk of harm through study participation. In many cases, the women and their pregnancies are scrutinized and followed carefully. Accurate and complete capture of perinatal outcomes is often essential to assessing the safety of interventions during pregnancy [4].

## 3. Study design

### 3.1. Sample size considerations

Studies are designed based on baseline data, collected through previous studies or public records. The prolonged process from grant writing to start of the study virtually ensures that baseline data will be outdated by the time the new clinical trial begins. This is true for designing clinical trials in all settings as secular and seasonal variations are the hallmark of communicable diseases. In resource limited settings, the added elements of sporadic and unpredictable availability of resources lead to changes in preventive strategies available in the general community. As an example, in our continuous surveillance of malaria prevalence in pregnant women and in communities, we have consistently found that a single bed net campaign may decrease malaria prevalence dramatically for one year and then return to the previous baseline level subsequently (Boudova and Laufer, unpublished data). Public records are also unreliable and inconsistent. Definitions that distinguish stillbirths from miscarriages and growth restriction from preterm birth require accurate antenatal assessment of gestational age, which is rarely available [5].

Another unique characteristic of research in the most resource-limited environments is the disparity between standard of preventive and curative treatment policies and the access that most women have to those treatments [1,6]. As discussed below, investigators are obligated to provide clinical trial participants with, at least, the basic care to which they are entitled. While this obligation is essential, when such services are not available to the population at large, such care alone will likely have an effect on the natural history of a wide range of infectious diseases and also the incidence of adverse perinatal and neonatal outcomes. A significant decrease in baseline rates of these key outcome measures can limit the power of clinical studies. In our studies, the provision of bed nets to prevent malaria is a key element of the antenatal care package, though the local government clinic frequently experience stock outs. Active detection and treatment of anemia, hypertension, urinary tract infections and sexually transmitted diseases that often does not occur in busy public clinics, likely improves the perinatal and infant outcomes among all participants.

### 3.2. Eligibility criteria

To ensure some uniformity in the study population, gestational age windows are specified in the eligibility criteria [2]. Assessment of gestational age of the pregnancy is typically performed by calculation based on last menstrual period or measurement of the fundal height. Even when implemented correctly, these techniques do not provide consistent results [7–9]. In our experience, women often do not recall their last menstrual period and busy midwives often do not have time to measure the fundal height or do not have measuring tapes. Visual inspection and palpation of the abdomen are used to give a rough estimate of gestational age. For a clinical trial, more precise measurements are required and the use of ultrasound dating is essential. Portable and inexpensive ultrasound machines are now available for use in resource-limited settings [10]. However, this capacity to accurately date pregnancies requires training and supervision as described below.

When participants are expected to be enrolled prior to the third trimester, recruitment may be difficult. Reaching women during the early stages of their pregnancy poses a challenge. There are social concerns about revealing ones pregnancy “too early”. Women typically present for their first antenatal visit late in their second or even in their third trimester [11–14], limiting the ability to capture data during early fetal development.

### 3.3. Follow up

The ability to maintain the follow up schedule through pregnancy has been identified previously as a barrier to obtaining adequate safety data [2]. Follow up fatigue often sets in. The World Health Organization recommends a minimum of four antenatal care visits. For active case detection, administration of interventions and monitoring for adverse events, participants are often asked to attend more antenatal visits than this commonly-accepted minimum. Although, transportation costs are reimbursed for participants at all scheduled visits, increased antenatal visits compete with other obligations for participants as well as the physical fatigue of pregnancy, all contributing to the risk of reduced adherence to follow up schedules over time.

There are local traditions that encourage women to deliver their infants at health facilities located close to their extended families. These customs are essential because family members provide all care for pregnant women and their newborns. Although, we only included women who agreed to deliver their infant at the study designated health center; we found that delivery plans changed over the course of the pregnancy. As the first antenatal visit coincided with the first public statement of the woman’s pregnancy, negotiations about details of the delivery, especially, for women who are pregnant for the first time, evolve over the subsequent months.

Changes in participation as a result of adverse events experienced during the study significantly threaten the integrity of the study. We have observed a wide range of responses. Most often, when complications related to pregnancy occur, participants are grateful to the study team members for the medical care, logistical support and advocacy they provide. Research clinicians and nurses are able to help navigate the often complex health system and provide care that is better than what is available through the typical public health infrastructure. However, adverse events, even when clearly unrelated to study intervention, often elicit suspicion and fear. As a result, women who experience complications of pregnancy either choose to discontinue study participation or withdraw due to pressure from family members who attribute the complication to research participation. In these cases, loss to follow up is strongly associated with pregnancy outcomes.

### 3.4. Detection of baseline illnesses and exclusion criteria

The eligibility criteria, especially, for trials of new interventions that may have unanticipated risks, are often strict. Potential participants undergo extensive evaluation, often well beyond the standard screening offered to women in the antenatal settings, to assess their eligibility for the study. Thus, women who would have underlying illnesses that would otherwise remain undetected at an early stage will be systematically excluded from the clinical trial. This is undoubtedly essential for the protection of the welfare of those who enroll. However, conclusions about safety and efficacy in a real life population are severely limited. Outcomes will be demonstrated in women with or without conditions, which were identified through screening tools that may never be avail-able in routine setting, so a conclusion, for example, that a drug is safe as long as a pregnant women do not have hypertension may not be relevant in the setting where blood pressure is not carefully monitored.

### 3.5. Capturing endpoints

Deliveries are unpredictable. They occur day and night, though typically more often in the night [15]. This trend has not been maintained recently in the United States [16], but likely remains true in resource-limited settings. They can occur in any location, not always at a health facility and certainly not at the previously identified health facility of choice. Capturing data from maternity wards is perhaps one of the most challenging of all health care environments. They are chaotic places where frequently a single nurse midwife is overseeing multiple women in labor and may be responsible for both maternal and neonatal care during the delivery. Women are discharged home quickly, with limited assessment of the infant. While birth weight is often recorded, its accuracy is not assured. Other key measurements such as height and head circumference may not be routinely recorded. Often temperature is not measured routinely so fever and hypothermia are usually not detected until it is severe.

## 4. Cultural, ethical and regulatory concerns

### 4.1. Consent

One of the basic tenets of research ethics is autonomy – individual’s capacity to deliberate about own goals and to act under the direction of such deliberation without manipulation by external forces [17]. Individual autonomy is more complex in settings where community and family structures have a strong influence on individual choices. This is a unique challenge to pregnant women as the decisions that affect the fetus often are perceived to lie with people other than the mother. Subpart B of 45 Code of Federal Regulations Part 46 clearly states that for studies in which the risk to the fetus is not greater than minimal, consent of just the pregnant woman is sufficient [18]. However, our experience is that this is not how decisions are made. Key influential family members, especially mother in law and other paternal matriarchs are often the decision makers. Although, consultation with family members does not negate individual autonomy, in fact is an exercise of it, this does present some unique challenges.

For studies that seek to enrol women at their first antenatal visit, the need to consult individuals who are not present during the visit is problematic. If randomization to an intervention vs. standard of care is meant to occur at the first antenatal visit, then a delay in provision of at least a standard intervention raises the concern that a potential participant will receive inadequate antenatal care while she is deciding whether or not to join the study. The period without the protection of standard antenatal intervention may increase the period of the pregnant woman’s vulnerability to infection and there is a risk that the woman will not return in a timely manner especially if she decides against study participation. Our staff routinely asks potential participants if there are other key decision makers with whom they would like to discuss their participation prior to enrolment. However, we have experienced substantial numbers of withdrawals of consent following first visits because of the input of influential family members. It is tempting to insist upon identifying and consulting key decision makers, however, this also disempowers the woman from making decisions.

### 4.2. Trust

Enrolment into a study depends on how much trust the potential participant has in the study and its staff. This trust is largely based on the expectation that the study will maximize good and minimize harm. With pregnancy, these expectations are held even more strongly with the anticipation of healthy outcomes and the intuitive understanding that bad outcomes can occur without warning. In a setting of extended families and strong community bonds, the study needs to gain the trust of not only the participants, but also their husbands, parents and traditional leaders. The community carefully scrutinizes any new activities that are related to pregnancy health care. Mistrust can develop quickly and can breed misconceptions that have the potential to derail study recruitment and follow up. In our study, the common misconceptions were that the study team collects human specimens including blood and placentas for profit gains or witchcraft. Other community members have claimed that study participation leads to poor pregnancy outcomes and infant death.

## 5. Generalizability

As it is clear from the above discussion, participants who attend antenatal care within the desired gestational age range, have family support and meet the strict inclusion and exclusion criteria, represent a unique subset of pregnant women. Through their participation in the clinical trial, they undergo screening and treatment for many infectious and non-communicable conditions that will alter pregnancy outcomes. The question then always arises – To what extent does the outcome of the clinical trial predict the impact of implementation of the intervention under real-life conditions?

## 6. Proposals to prevent and mitigate

### 6.1. Partnering with local health care providers

The local health care facilities providing maternal care can play a pivotal role in recruitment of participants and tracking of delivery outcomes. In our studies at a local health center, nurse midwives from these facilities were trained on the specifics of the studies, including participant recruitment, identification of a study participant in labor and collection of delivery outcomes. Though there is inherent tension between government and study personnel and assigned duties and responsibilities, the local study team focused intensely on maintaining a collegial and cooperative relationship with the local health center staff.

Another level of partnership was with community health care workers (CHWs), whose formal job is implementation of public health interventions at community level and are often consulted by community members on various health topics. The CHWs were trained on the details of the clinical studies, the screening process and basics of what potential participants should expect if they choose to join the study. Their involvement was instrumental in engaging in a strong working relationship with the community. They also serve as trusted advisors to community members and especially appeal to individuals who have some inherent distrust of the government health infrastructure. Thus, in addition to facilitating communication, they also helped the study to gain acceptance.

### 6.2. Community engagement

To maintain strong lasting ties with the community, the site, with the help of the research ethics committee, established a community advisory group, a committee of volunteers from the study catchment area trained on the basics of clinical trials, research ethics and the specifics of ongoing studies. The group represents the interests of the community with regard to ongoing and new studies. It also acts as a liaison between the research team and the community, educating people about participation in clinical trials and providing a platform of ongoing communication between the research team and the community.

Because of the reluctance to reveal pregnancy early, it is difficult to encourage discussion about pregnancy related issues prior to initiating antenatal care. There is no specific target audience for women who are pregnant but have not reached antenatal care, the fathers of their children and their parents. Information must reach the entire community with the hope that in the short period between revealing pregnancy and attending the first antenatal clinic visit, study participation is discussed.

To reach out to men, parents and others with influence over women’s participation in clinical studies that are otherwise not accessible because they do not attend antenatal clinics, site staff conducted community meetings around the catchment area. To attract these key decision makers, the activities were organized in a formal way through community leaders and included performance by a local drama group. The activities targeted common misconceptions about study participation and pregnancy outcomes and accommodated questions from community members to address their concerns.

### 6.3. Basic use of ultrasound

The use of ultrasound for dating pregnancies prior to the third trimester is an attainable goal. Portable, rugged ultrasound machines are available for use in resource limited settings for a wide range of obstetric and non-obstetric uses. We have successfully established on-site ultrasound capacity for gestational age dating in the second trimester at our research clinic in Malawi [19]. A US trained obstetrician spent one week in person training key staff members in the technique. For four months, the US expert reviewed all scans and provided real-time feedback. With the intense quality control procedures, several site staff became highly skilled in this specific activity. They subsequently trained new staff members and served as their mentors as the new trainees began to conduct the scans themselves. Today, we conduct 10–15% quality assurance on all scans to ensure that the quality remains high. The endeavor, however, is not trivial.

### 6.4. Providing benefits to early enrolment in antenatal care

In the absence of interventions that get at the very deep seeded reluctance among women to identify themselves as being pregnant [20–22], clinical studies can offer incentives to early enrolment in antenatal services. One obvious benefit in studies is the relief of the costs of antenatal care to the individual woman. In this way, one of the leading barriers to antenatal care attendance, lack of economic means to register for care [12,23,24], can be overcome. With this support, the first antenatal visit may be perceived as less of an investment and thus a decision that can be made earlier in gestation.

The ultrasound images are also novel and often highly desirable benefits of study participation and potentially incentive to seek out antenatal care. If there are limited tangible benefits to enrolment in antenatal care and the care itself does not provide adequate reassurance about the viability of the pregnancy, a live image of the fetus and a picture to take home to show to family members has been strongly appreciated by study participants. Investigators in other Africa settings have had similar anecdotal experience [14,25,26] and a clinical trial is currently underway to assess the benefit of routine ultrasounds in resource-limited settings with the rigor of a randomized trial [27].

One unanticipated benefit was the ability to conduct urine pregnancy tests. This capacity was not available at the government clinic from which we recruited study participants. Young women in their first pregnancy occasionally came to the government clinic to ascertain whether they were pregnant and through the standard care, pregnancy would be diagnosed by history of last menstrual period and palpation, which was often indeterminate early in pregnancy. Women who were interested in being screened for study participation were offered urine pregnancy test if pregnancy was not clear from physical examination. These women were grateful for the service provided by the study team and were eager study participants when they were enroled.

### 6.5. Flexibility and re-evaluation

Because of the unpredictable nature of communicable diseases and the potential of unanticipated and sometime unmeasurable factors that influence the local epidemiology, sample size and event rate calculations must allow for some flexibility. Changes in the incidence of disease and adverse outcomes should be anticipated and opportunities for re-evaluation should be built in to protocol time lines. Sponsors should also be prepared to support changes in these parameters when unpredictable fluctuations occur.

### 6.6. Advocacy

Researchers from the local institutions and from abroad are in a unique position of power. They often develop close working relationships with public health leaders, make meaningful contributions to the health care provided in the community where they work and help to translate scientific discoveries into public health action. By functioning in close association with the public health system, investigators experience first-hand the challenges faced by local health centers. We therefore have an opportunity and even a responsibility to advocate on behalf the communities we serve. There is a selfish view of this – by making the access to healthcare in “real life” more similar to the conditions of clinical trials, we improve the generalizability of our results – but we believe we have a moral obligation. Communities trust that researchers are working to improve their health. While researchers think of this role as contributing new scientific knowledge, we can and should have an obligation work to make the best possible health care today available to those communities who are willingly volunteering to improve the health of those who come after them.

## 7. Conclusion

Conducting clinical trials among pregnant women in resource limited settings presents unique epidemiological and ethical challenges including limited availability of baseline data, the quality of standard of care when compared to international standards, unpredictable changes in disease epidemiology and cultural beliefs. In our decade of leading clinical studies of pregnant women in Malawi, we have found that partnering the local health care system, community engagement, incentivizing study participation, and adding flexibility to study designs are essential to maintaining a successful program.
